# Simultaneous study of behavioral synchronization of two individuals during a cooperative task

**DOI:** 10.1192/j.eurpsy.2022.2241

**Published:** 2022-09-01

**Authors:** B. Kakuszi, M. Fullajtar, I. Bitter, P. Czobor

**Affiliations:** Semmelweis University, Psychiatry And Psychotherapy, Budapest, Hungary

**Keywords:** mentalization, behavioral synchronization, social neuroscience, psychiatric disorders

## Abstract

**Introduction:**

Interpersonally coordinated behaviors are crucial for social interactions.The “Theory of Mind,” or mentalization capacity, of an individual is essential for the establishment of behavioral synchronization. The Reading the Mind in the Eyes Test (RMET) is used to assess mentalization, social cognition and empathy. Previous RMET studies, investigated people in isolation, not in social situations. It is unclear how the RMET predicts functioning during real-life social interactions.

**Objectives:**

To investigate the relationship between the performance measured on the RMET test and the synchronous behavior of two individuals interacting with each other during tasks requiring social collaboration.

**Methods:**

Sample included healthy controls (HC,n=48) and patients with ADHD (n=26) or schizophrenia (SCH,n=36) from an ongoing EEG-hyperscanning study, employing a social coordination condition.We applied a Go/NoGo reaction time(RT) task performed by pairs of participants. Synchronous behavior was characterized by the correlation of participants’ RTs.We used the percent (%) correct responses from the RMET to characterize social cognition.

**Results:**

In HC, with better social cognitive performance, the correlation of behavioral responses was significantly (p<0.05) higher. In ADHD, better performance on the RMET was also accompanied by better behavioral synchronization, but the association did not reach significance due to the smaller sample size. In SCH, no relationship was detected.

**Conclusions:**

In HC and ADHD, the mentalization ability as measured by RMET is associated with the behavioral synchronization between individuals in social interaction.The lack of association in the schizophrenia group may be due to psychopathological symptoms, which should be elucidated in future research. Funding: Supported by the Hungarian Brain Research program#2017-1.2.1-NKP-2017-0002

**Disclosure:**

No significant relationships.

